# Species Deletions from Microbiome Consortia Reveal Key Metabolic Interactions between Gut Microbes

**DOI:** 10.1128/mSystems.00185-19

**Published:** 2019-07-16

**Authors:** Natalia Gutiérrez, Daniel Garrido

**Affiliations:** a Department of Chemical and Bioprocess Engineering, School of Engineering, Pontificia Universidad Catolica de Chile, Santiago, Chile; Duke University School of Medicine

**Keywords:** bioreactor, keystone species, microbiome, butyrate, metabolic interaction

## Abstract

The gut microbiome is a complex microbial community that plays a key role in human health. Diet is an important factor dictating gut microbiome composition. This is mediated by multiple microbe-microbe interactions that result in the fermentation of nondigestible carbohydrates and the production of short-chain fatty acids. Certain species play key metabolic roles in the microbiome, and their disappearance could result in dysbiosis. In this work, a synthetic consortium of 14 gut microbes was studied during the utilization of prebiotic inulin in batch bioreactors. Fermentations were repeated leaving one species out every time, in order to evaluate the impact of their elimination on the system. Substrate consumption, microbial composition, and metabolite production were determined. Single deletions never resulted in a complete loss of bacterial growth or inulin consumption, suggesting functional redundancy. Deletions of Bacteroides dorei and Lachnoclostridium clostridioforme resulted in lower biomass and higher residual inulin. The absence of *B. dorei* impacted the abundance of the other 10 species negatively. Lachnoclostridium symbiosum, a butyrate producer, appeared to be the most sensitive species to deletions, being stimulated by the presence of Escherichia coli, Bifidobacterium adolescentis, *B. dorei*, and Lactobacillus plantarum. Conversely, bioreactors without these species did not show butyrate production. *L. clostridioforme* was observed to be essential for propionate production, and *B. dorei* for lactate production. Our analysis identified specific members that were essential for the function of the consortium. In conclusion, species deletions from microbial consortia could be a useful approach to identify relevant interactions between microorganisms and defining metabolic roles in the gut microbiome.

**IMPORTANCE** Gut microbes associate, compete for, and specialize in specific metabolic tasks. These interactions are dictated by the cross-feeding of degradation or fermentation products. However, the individual contribution of microbes to the function of the gut microbiome is difficult to evaluate. It is essential to understand the complexity of microbial interactions and how the presence or absence of specific microorganisms affects the stability and functioning of the gut microbiome. The experimental approach of this study could be used for identifying keystone species, in addition to redundant functions and conditions that contribute to community stability. Redundancy is an important feature of the microbiome, and its reduction could be useful for the design of microbial consortia with desired metabolic properties enhancing the tasks of the keystone species.

## Abstract (precis)

Gut microbes associate, compete for, and specialize in specific metabolic tasks. These interactions are dictated by the cross-feeding of degradation or fermentation products. However, the individual contribution of microbes to the function of the gut microbiome is difficult to evaluate. It is essential to understand the complexity of microbial interactions and how the presence or absence of specific microorganisms affects the stability and functioning of the gut microbiome. The experimental approach of this study could be used for identifying keystone species, in addition to redundant functions and conditions that contribute to community stability. Redundancy is an important feature of the microbiome, and its reduction could be useful for the design of microbial consortia with desired metabolic properties enhancing the tasks of the keystone species.

## INTRODUCTION

The gut microbiome is a complex microbial community characterized by a high cell density, reaching numbers similar to somatic cells (1). Dominant phyla are *Firmicutes* and *Bacteroidetes*, which could reach up to 90% of the total bacteria (2). Other phyla with minor but significant representation are *Proteobacteria*, *Actinobacteria*, and *Verrucomicrobia* (2).

The impact of dietary substrates on the gut microbiome is one of the most remarkable and best studied (3, 4). Certain components not absorbed in the small intestine reaching the colon, such as complex carbohydrates and partially degraded proteins, are accessed by gut microbes. Some of these substrates are considered prebiotics and are of great interest in order to modify the gut microbiome for promoting health (5, 6). Fermentation of these macromolecules results in the production of short-chain fatty acids (SCFA), such as acetate, propionate, and butyrate (7). These molecules display several physiological effects on the host (8, 9), such as influencing the stability and physiology of gut environment and serving as an energy source for colonocytes (10). These SCFA represent up to 90% of total acids and have an approximate molar ratio ranging from 75:15:10 to 40:40:20, respectively (11, 12). Among several roles, butyrate helps to maintain the integrity of the mucosa, protecting against cellular inflammation and promoting the removal of dysfunctional cells (13, 14).

The influence of diet on gut microbiome composition and diversity is probably mediated by metabolic interactions between gut microbes (15, 16). These interactions are the basis of complex ecological networks, characterized by cooperative and competitive relationships (17). These could in turn modulate the activity and stability of the gut microbiome. Gut microbes play distinct metabolic roles specializing in the degradation of complex polysaccharides, the fermentation of simple monomers, or the production of essential metabolic intermediates (16, 18). Cross-feeding is a strong force guiding microbiome composition, where certain intermediates produced or released by one microbe are utilized by another. These metabolites derive from the degradation of complex macromolecules, resulting in simple monomers, or from the production of fermentation products such as SCFA or amino acids (19).

The metabolic functions assigned to the gut microbiome are remarkably more conserved than microbial composition, suggesting redundant functionalities (20). However, microbial diversity in the gut microbiome has been shown in several studies to be an important parameter associated with health. In the gut microbiome, certain species have been shown to display a large impact on the structure and function of the community (21). These keystone species could produce unique metabolites connecting two populations or providing essential metabolites for the host. Therefore, they have a crucial role in maintaining the organization of communities due to their functional capabilities and their biotic interactions with other members of the community (22, 23).

Conditions resulting in the loss of keystone species could lead to a dysbiotic state that impairs the integrity of the gut ecosystem (24, 25). Dysbiosis has been linked to certain disorders such as atopic dermatitis and obesity (26) and inflammatory bowel diseases (IBD) (27). Studies have associated IBD with a decrease in the abundance of butyrate-producing species such as Faecalibacterium prausnitzii and *Roseburia* sp. (10, 28). Interestingly, their reincorporation into the microbiome restores the production of butyrate and consequently promotes the recovery of gut homeostasis (28). This highlights their pivotal importance in the stability and function of the gut microbiome.

To understand the role of the gut microbiome in health and even target the microbiome as a therapeutic target, it is important to define better the metabolic roles performed by gut microbes and their metabolic interactions (29). Recent studies have shown that paired cocultures of gut microbes could explain the behavior of more-complex communities (17, 30). However, it is not well known how individual species could affect the stability and performance of larger microbiome consortia. In this study, we evaluated a species deletion approach to synthetic microbiome consortia, in order to evaluate the impact of single species on the microbial composition and the metabolic function of the microbial community.

## RESULTS

### General properties of the microbial consortium.

**FIG 1:**
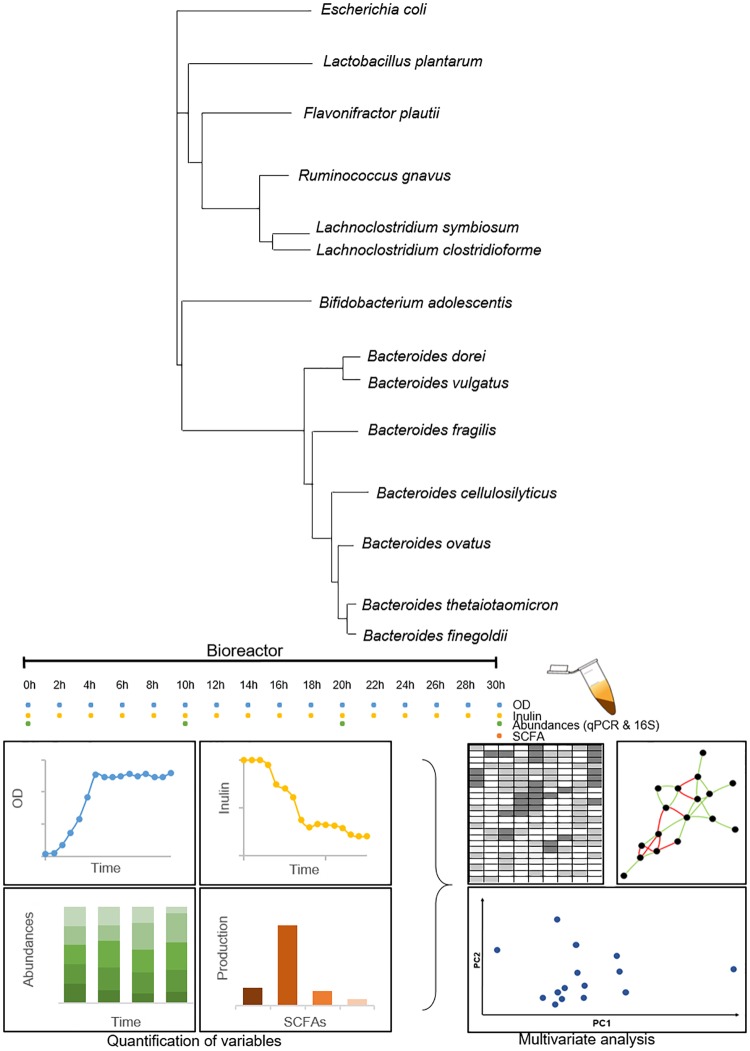
Microorganisms in the synthetic consortium and setup of the study. Distance tree indicates genetic distances between 16S rRNA sequences of the microorganisms. The colored circles indicate the samples analyzed for the quantification of variables in the bottom right. Blue and yellow circles indicate OD and inulin consumption determinations, respectively, and green circles indicate microbial composition analysis. The orange circle indicates total SCFA quantification at the end of each experiment.

The setup of the study is shown in Fig. 1 We included 14 microorganisms frequently found in the gut microbiome (31–33), which belong to the *Firmicutes* (5 species), *Bacteroidetes* (7 species), *Proteobacteria*, and *Actinobacteria* (Fig. 1). All microorganisms had their genomes sequenced (Table 1). Genome comparison with other strains in the same species indicated that the selected microorganisms were in general representative of their species, with average nucleotide identity values higher than 97% compared to other strains (see data sets at https://figshare.com/s/a3c67977ccf6fe7292eb). Some species had previous evidence for inulin utilization or are endowed with fructofuranosidases (Table 1). The encoded potential for SCFA production was deduced from their genomes (Table 1), indicating that the consortium was able to produce lactate, acetate, propionate, or butyrate. Two species had the potential to produce butyrate: Lachnoclostridium symbiosum and Lachnoclostridium clostridioforme.

**TABLE 1:** Description of the strains used in this study and genomic features important for the study

Phylum, species, and strain	Genome size (Mbp); %GC	Inulin utilization and fructofuranosidase (locus tag[s])	SCFA production				Culture medium^*a*^	Sporulation	Reference(s)
			Lactate	Acetate	Propionate	Butyrate			
*Firmicutes*									
Lactobacillus plantarum ATCC 8014	3.23; 44.53	Yes; Ga0133059_101166	Yes				MRS		50
Ruminococcus gnavus CC55_001C	3.18; 43.10	No; HMPREF1201_02241, HMPREF1201_01812, HMPREF1201_01164	Yes	Yes			RCM	Yes	67
Flavonifractor plautii 1_3_50AFAA	4.32; 60.53	No				Yes	RCM	Yes	68
*Lachnoclostridium* *symbiosum* WAL-14673	4.86; 48.17	No; HMPREF9475_00225				Yes	RCM	Yes	55
*Lachnoclostridium* clostridioforme 2_1_49FAA	5.46; 48.93	No; HMPREF9467_03179		Yes			RCM	Yes	69

*Bacteroidetes*									
Bacteroides dorei 5_1_36/D4	5.53; 41.52	Yes; BSEG_04148	Yes	Yes	Yes		BHI		48
Bacteroides vulgatus ATCC 8482	5.16; 42.20	No		Yes	Yes		BHI		53
Bacteroides finegoldii CL09T03C10	5.12; 42.50	No; HMPREF1057		Yes	Yes		RCM		53
Bacteroides fragilis CL03T12C07	5.21; 43.44	Yes; HMPREF1067_02001	Yes	Yes	Yes		BHI		70
Bacteroides ovatus 3_8_47FAA	6.55; 41.97	Yes; HMPREF1017_04501		Yes	Yes		BHI		53
Bacteroides cellulosilyticus CL02T12C19	7.68; 43.05	No; HMPREF1062_02875	Yes	Yes	Yes		BHI		53, 71
Bacteroides thetaiotaomicron VPI-5482	6.29; 48.86	Yes; BT1760	Yes	Yes	Yes		RCM		72

*Proteobacteria*									
Escherichia coli K-12 MG1655	4.64; 50.79	No	Yes	Yes			LB/PCA		73

*Actinobacteria*									
Bifidobacterium adolescentis ATCC 15703	2.09; 59.18	Yes; BAD_1150, BAD_1325	Yes	Yes			MRS-Cys		74

a MRS, de Man, Rogosa, and Sharpe medium; PCA, plate count agar.

### Reproducibility of the culture system.

We first evaluated the reproducibility of the culture system, culturing the 14 microorganisms in a batch bioreactor using inulin as carbon source. Microorganisms were inoculated at similar initial concentrations. Culturing the consortium with all species (designated All) showed a coefficient of variation (CV) of 3.3% in optical density (OD) and only 3.4% in the final inulin amount left (see Fig. S1A at https://figshare.com/s/a3c67977ccf6fe7292eb). The relative abundances of each species showed an average CV of 18.6% in the final composition of the replicates (see Fig. S1B at https://figshare.com/s/a3c67977ccf6fe7292eb). The initial composition between the bioreactors showed an average CV of 8.1% (see Fig. S1C at https://figshare.com/s/a3c67977ccf6fe7292eb). These results indicate that the bioreactor is a reproducible culture system for the purpose of evaluating the impact of species deletions.

### Bacterial growth.

**FIG 2:**
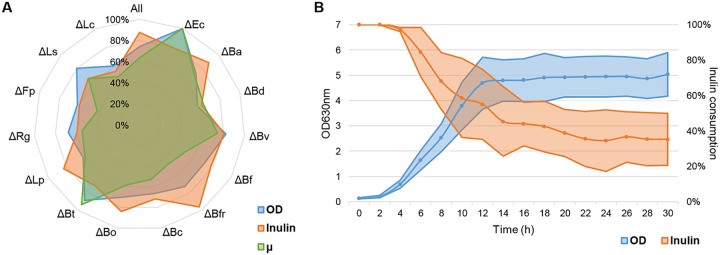
Growth (maximum OD and growth rate [μ]) and inulin consumption in deletion experiments. (A) Comparative relationship between growth parameters of each bioreactor (see key). Each parameter was normalized to the maximum value observed between experiments (100%). Deleted species are denoted after each triangle: Ec, E. coli; Ba, *B. adolescentis*; Bd, *B. dorei*; Bv, B. vulgatus; Bf, *B. finegoldii*; Bfr, B. fragilis; Bc, *B. cellulosilyticus*; Bo, *B. ovatus*; Bt, B. thetaiotaomicron; Lp, *L. plantarum*; Rg, *R. gnavus*; Fp, *F. plautii;* Ls, *L. symbiosum*; Lc, *L. clostridioforme*. (B) Average of growth (blue) and inulin consumption (orange) values of all bioreactors. Shaded areas represent the standard deviation range of each time point.

Microbial growth, growth rate, and inulin utilization were determined for each bioreactor after every species deletion (Fig. 2). Every deletion experiment was performed once. The exponential phase started between times 4 and 6 h, and all bioreactors reached stationary phase between 12 and 14 h (Fig. 2B; see also Fig. S2 at https://figshare.com/s/a3c67977ccf6fe7292eb). Escherichia coli and Bacteroides thetaiotaomicron deletions resulted in the highest biomass and growth rates obtained, indicating that these microorganisms contribute negatively to these parameters in the whole consortium (Fig. 2). In contrast, when leaving Bacteroides dorei and Lachnoclostridium clostridioforme out of the consortium, we observed less biomass and a lower growth rate, suggesting they are important for the growth of the consortium. Their deletion could therefore free carrying capacity of the system. Considering all dropouts, the maximum deviation between each bioreactor was an OD of 1 relative to the average (Fig. 2B). The maximum OD variation between deletion experiments was not very high; it went from 4.23 (*B. dorei* deletion) to 7.36 (E. coli deletion; see Fig. S2 at https://figshare.com/s/a3c67977ccf6fe7292eb). In aggregate, these results indicate that deletion of individual species does not dramatically affect microbial consortia in terms of total growth. However, deletion of certain species resulted in certain alterations increasing or decreasing the biomass of the system.

### Inulin consumption.

Substrate consumption showed variations of up to 40% between bioreactors (Fig. 2B). An almost complete inulin consumption was observed after deleting Bacteroides fragilis (see Fig. S2 at https://figshare.com/s/a3c67977ccf6fe7292eb). Deletion of this microorganism resulted in slower growth compared to all other replicates (Fig. 2A). These results indicate that B. fragilis in this system interferes with inulin consumption. The opposite was observed in *L. clostridioforme*, B. thetaiotaomicron, and *B. dorei* deletion bioreactors, where the absence of these microorganisms resulted in an important amount of residual inulin at the end of the fermentation (see Fig. S2 at https://figshare.com/s/a3c67977ccf6fe7292eb). This indicates that either directly or indirectly, they are critical in the consumption of the prebiotic. In summary, this analysis identified certain microorganisms important for inulin utilization in the consortium and also one member that negatively impacted substrate consumption. Interestingly, none of the members of the consortium appeared to be irreplaceable regarding inulin consumption, since all bioreactors displayed fast growth and utilized at least 40% of the inulin. This suggests functional redundancy in the metabolism of the consortium (Table 1).

### Microbial composition of bioreactors.

**FIG 3:**
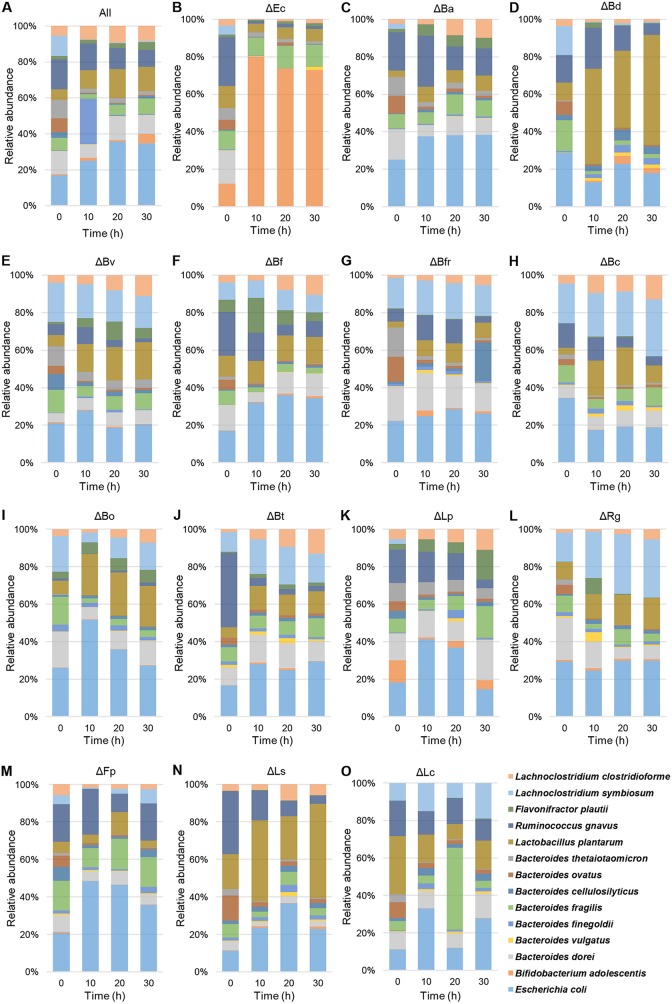
Relative bacterial abundances in every bioreactor during growth in inulin at 0, 10, 20, and 30 h. Cell copy numbers were determined by qPCR and were expressed as a proportion of the total number of cell copies in each time point in each bioreactor. Colors at the bottom indicate the relative abundance of each microorganism. Bar plots show the relative microbial composition of the complete consortium (A) or after the deletion of the microorganism indicated in the center at the top (B to O). See Fig. 2A legend for definitions of organism abbreviations.

The microbial composition of each consortium was determined by quantitative PCR (qPCR) (Fig. 3). Cell copy numbers were in general correlated with OD values (see Fig. S3 at https://figshare.com/s/a3c67977ccf6fe7292eb). In general, the dominant species were Ruminococcus gnavus, E. coli, *B. dorei*, *L. symbiosum*, and Lactobacillus plantarum. *L. plantarum* showed a considerable increase in the absence of *B. dorei* and *L. symbiosum*, reaching 50% of the total abundance and suggesting a competitive relationship between these microbes (Fig. 3D and N). Bifidobacterium adolescentis was not particularly dominant in any bioreactor, which was unexpected considering that it is a prominent inulin utilizer (34, 35). One remarkable exception was the E. coli deletion bioreactor, where *B. adolescentis* increased its relative abundance to more than 60% (Fig. 3B).

**FIG 4:**
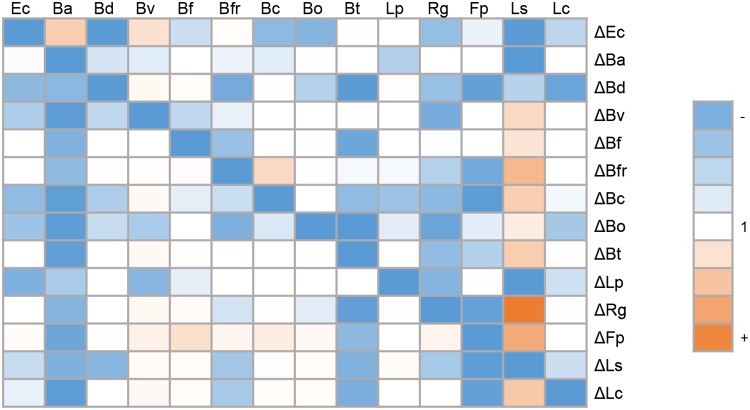
Effect of the absence of a strain on the abundance of the remaining microorganisms in the consortium. Colored cells indicate the abundance ratio of each bacterial species (top) between a deletion experiment (right) and the abundance of the same microorganism in the All bioreactor. A ratio of 1 is represented in white. Orange boxes indicate that the strain increased its abundance in the bioreactor indicated to the right with respect to the control, and blue boxes indicate a decrease in abundance with respect to the control (All bioreactor). See Fig. 2A legend for definitions of organism abbreviations.

To quantify the effect of the absence of one microorganism on the abundance of other species in the consortium, an abundance ratio was calculated for each deleted microorganism relative to the abundance of the same strain in the All bioreactor (Fig. 4). Deletions of *B. adolescentis* and Bacteroides finegoldii from the consortium did not alter the abundance of any other species. While Bacteroides vulgatus and *B. finegoldii* were mostly unaffected by any dropout, *B. adolescentis* and Flavonifractor plauti showed reduced abundances in the absence of almost any other member. Finally, deletion of *B. dorei* from the consortium caused the most deleterious effect on the abundance of 10 microorganisms (Fig. 4), indicating a key role of *B. dorei* in this consortium.

Certain species benefited from the absence of others. The abundance of *L. symbiosum* was stimulated in the absence of eight species of the consortium but also negatively impacted by the absence of four others (E. coli, *B. adolescentis*, *B. dorei*, and *L. plantarum*). It could be hypothesized that the latter four are important for *L. symbiosum* growth since their removal impacted *L. symbiosum* abundance negatively. This also indicates that *L. symbiosum* is the most sensitive member relative to the presence or absence of other microorganisms in this consortium.

### Production of metabolites.

**FIG 5:**
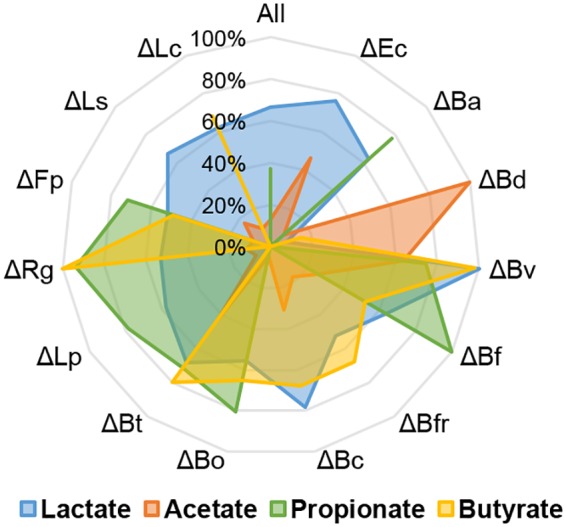
Production of SCFA in every bioreactor, quantified by HPLC. The concentration of each acid (key at the bottom) was normalized relative to the maximum value of each SCFA determined (100%). SCFA were quantified at 30 h of each experiment from bacterial supernatants. See Fig. 2A legend for definitions of organism abbreviations.

The concentrations of lactate, acetate, propionate, and butyrate were determined at the end of each run and normalized to the maximum value observed (Fig. 5; see also Fig. S4 at https://figshare.com/s/a3c67977ccf6fe7292eb). The production of SCFA in the All consortium was 83.9 mM lactate, 21.5 mM acetate, 5.9 mM propionate, and no butyrate.

The maximum production of lactate was reached in the B. vulgatus deletion bioreactor. Deletion of this microorganism also allowed the bioreactor to achieve the highest total SCFA concentration (see Fig. S4 at https://figshare.com/s/a3c67977ccf6fe7292eb). In contrast, deletion of *B. dorei* resulted in the minimal lactate concentration observed, concomitant with the maximum production of acetate observed. This indicates that *B. dorei* is essential for lactate production, and when it is absent, the consortium switches to high acetate production.

The maximum propionate concentration was observed in the *B. finegoldii* deletion bioreactor (16.1 mM), and the concomitant loss of six species did not result in the production of propionate (Fig. 5). Three of these were propionate-producing *Bacteroides* species (*B. dorei*, B. fragilis, and Bacteroides cellulosilyticus). Three others (E. coli, *L. symbiosum*, and *L. clostridioforme*) could contribute indirectly to propionate production.

**FIG 6:**
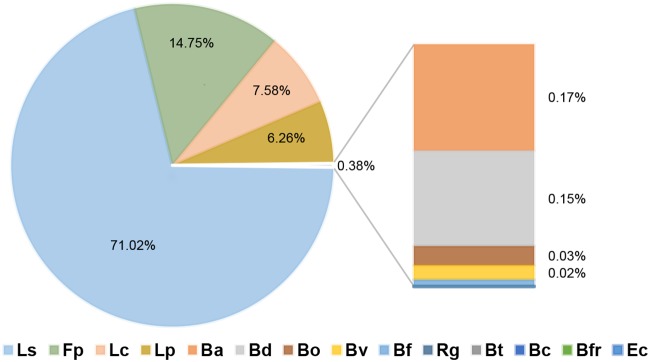
Percent contribution of each species to the butyrate production. The estimated contributions represent a linear relationship between the abundance of the species and butyrate production in each bioreactor. See Fig. 2A legend for definitions of organism abbreviations.

Finally, the higher concentration of butyrate was observed in *R. gnavus* and B. vulgatus deletion bioreactors (10.5 mM [Fig. 5]; see also Fig. S4 at https://figshare.com/s/a3c67977ccf6fe7292eb). This suggests that their presence has a negative impact on the production of the acid. In contrast, deletions of *L. symbiosum*, E. coli, *B. adolescentis*, *B. dorei*, and *L. plantarum* resulted in a lack of butyrate. A linear regression analysis of butyrate production and microbial abundance indicated that the major contribution to the production of this SCFA was attributed to *L. symbiosum* (Fig. 6). However, the abundance of other species such as *F. plautii*, *L. clostridioforme*, and *L. plantarum* also showed a positive correlation with the quantity of butyrate produced (Fig. 6).

### Correlation of variables.

**FIG 7:**
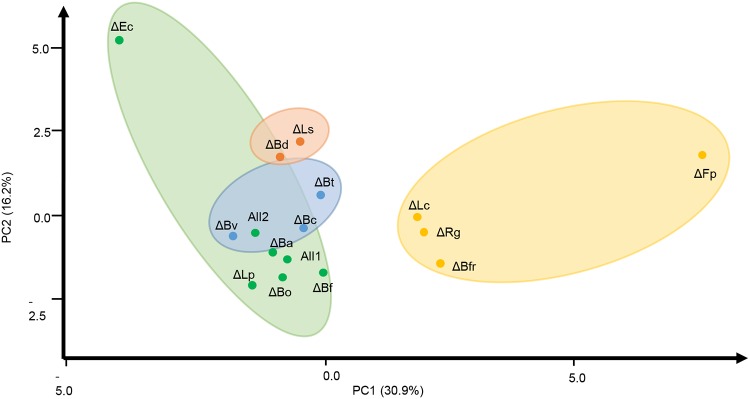
Principal-component analysis and clustering of consortia. Data analysis included as variables microbial species abundances, SCFA production, inulin consumption, OD values, and growth rate at the final point (*t* = 30 h). Colors represent the clusters formed by the deletion of each microorganism. See Fig. 2A legend for definitions of organism abbreviations.

To integrate these observations, a multivariate analysis was performed with all the variables described previously. A principal-component analysis showed four distinct clusters between the species deleted in each bioreactor (Fig. 7; see also Fig. S5 at https://figshare.com/s/a3c67977ccf6fe7292eb). These patterns could indicate functional similarities between these species. The cluster containing the All bioreactors included species which did not show much deviation from this control (*B. adolescentis*, *B. finegoldii*, *L. plantarum*, and Bacteroides ovatus), in addition to E. coli. A second cluster contained mostly *Bacteroides* species (B. vulgatus, *B. cellulosilyticus*, and B. thetaiotaomicron). Another cluster contained mostly *Firmicutes* species, which were important for SCFA production (*F. plautii* and *L. clostridioforme*) or controlled inulin utilization (B. fragilis). A fourth cluster was obtained with *B. dorei* and *L. symbiosum*, where their deletions from the system resulted in comparable results. Similar observations were also obtained when considering all data points (see Fig. S5 at https://figshare.com/s/a3c67977ccf6fe7292eb), where all bioreactors clustered closely at the beginning of the study, and most of the above associations were also found, including additional data points.

While the absence of *B. dorei* altered several parameters of the community, *L. symbiosum* abundance was essential for butyrate production (see Fig. S6 at https://figshare.com/s/a3c67977ccf6fe7292eb) and sensitive to the absence of several microorganisms. Finally, the abundance of *L. clostridioforme* was positively correlated with propionate production, that of *L. symbiosum* was positively correlated with butyrate generation, and that of *L. plantarum* was positively correlated with acetate production (see Fig. S6 at https://figshare.com/s/a3c67977ccf6fe7292eb).

**FIG 8:**
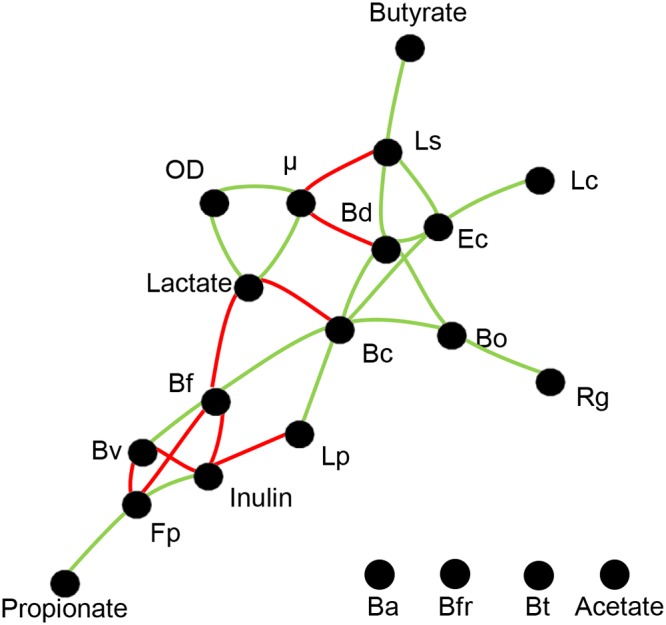
Interaction network from the correlation matrix between species abundances, SCFA production, and growth variables (inulin consumption, OD, and μ). These variables were obtained at the end of each run and represented by black dots. Red lines represent negative interactions, and green lines indicate positive interactions. Distance between nodes shows the intensity of the correlation between variables (the closer the dots, the stronger the interaction). See Fig. 2A legend for definitions of organism abbreviations.

We finally built an interaction network derived from a Spearman correlation matrix (Fig. 8). This network shows only statistically significant relationships between the variables (*P* < 0.05). Several negative correlations between microorganisms and parameters were found (Fig. 8). As expected, the butyrate production was correlated with *L. symbiosum*. Interestingly, *L. symbiosum* presence was positively correlated with only two species, E. coli and *B. dorei*. The presence of these microorganisms also formed a network of positive interactions with *L. clostridioforme*, *B. cellulosilyticus*, *B. ovatus*, *L. plantarum*, and *R. gnavus*. The abundances of *B. adolescentis*, B. fragilis, and B. thetaiotaomicron and acetate production were not significantly correlated with any other variable. After applying a correction of *P* values by multiple testing, only the interaction between *L. symbiosum* and butyrate and the interaction between E. coli and *B. dorei* remained significant.

**FIG 9:**
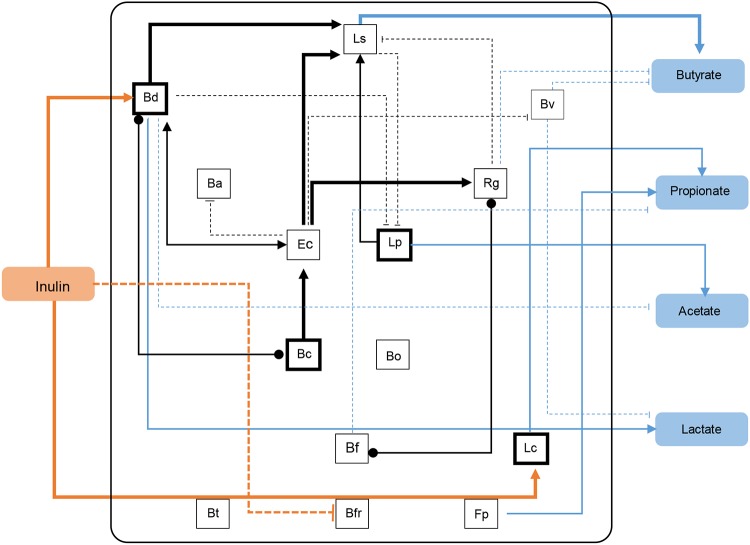
Working model of the study summarizing major results. Arrows indicate positive relationships. Dashed lines indicate negative relationships, and lines with points indicate a relationship with positive or negative status unknown. Thick lines indicate that these observations were statistically significant. Orange lines show the relationship between inulin and species that consume it, and blue lines show the relationship between species and the production of SCFA. See Fig. 2A legend for definitions of organism abbreviations.

Finally, Fig. 9 summarizes some of the findings of this study. A strong positive interaction was observed between *B. dorei* and *L. symbiosum*, which appeared essential for the function of the system converting inulin into SCFA, especially butyrate. *L. clostridioforme*, E. coli, and *L. plantarum* were species that with a minor contribution appeared supporting the function of *B. dorei* and *L. symbiosum*. Certain members of the consortium appeared to be unimportant (B. fragilis, B. thetaiotaomicron, and *F. plautii*), and a few appeared to contribute negatively to certain parameters (*B. finegoldii*, B. vulgatus, and *R. gnavus*).

## DISCUSSION

### Microbiome complexity and ecological interactions.

There is great interest in understanding the forces dictating gut microbiome composition and how the loss of the stability in the gut microbiome contributes to disease. This represents a great challenge, considering that the gut microbiome is composed of hundreds of species and multiple microbe-microbe and host-microbe interactions (36). One unifying principle is that this microbial community is highly responsive to dietary substrates arriving at the colon. This indicates that the chemical nature of dietary substrates being accessed by gut microbes dictates microbial metabolism and microbial interactions, resulting in complex cross-feeding networks. In this study, we analyzed the impact of species deletions on the composition and metabolic function of a synthetic consortium composed of 14 gut microbes during consumption of inulin. Every deletion experiment was performed once, considering that batch bioreactors showed to be reproducible. However, coculture experiments have shown that gut microbe growth could be very sensitive to the initial concentrations of microorganisms (37). Therefore, the lack of replicates is a limitation of this work.

Species deletion is a common approach in ecological theory of food webs (38, 39). Sometimes, the disappearance of one species in a biological community has detrimental effects on the whole community, causing further extinctions of species and impacting ecosystem performance and fitness (40). Interestingly, here we never found a species deletion causing a catastrophic effect in the community: total growth in the bioreactors always reached OD values higher than 4 and inulin utilization was always higher than 50% of the initial concentration. One possible explanation is that medium composition is directly responsible for the failure to observe larger metabolic dependencies between gut microbes. Considering that the substrate was readily consumed, another explanation is functional redundancy in this consortium, where the role of one species was readily replaced by another, leading to comparable final states. Redundancy is a crucial property of the adult gut microbiome, which has been directly associated with microbiome resilience and stability against perturbations (41).

In contrast to these similarities, microbial compositions and metabolic activities were markedly different after deletions of single microorganisms (Fig. 5; see also Fig. S4 at https://figshare.com/s/a3c67977ccf6fe7292eb). These observations are important since it is actually the metabolic activity of the gut microbiome, rather than the composition or taxonomy, which has the most profound effect on host physiology (42). *B. dorei* was the microbe that when absent caused the most substantial change in the consortium: 10 species showed a decrease in their relative abundance when *B. dorei* was missing. Concomitantly, total biomass was the lowest of all bioreactors, and a clear switch in SCFA was observed. Therefore, *B. dorei* could be classified as a keystone species in this consortium (40).

Interestingly, metabolic profiles did not necessarily reflect the metabolism of the dominant microbe in each deletion experiment. The deletion of *L. plantarum* resulted in low acetate concentrations, also showing a positive correlation between the abundance of *L. plantarum* and acetate level. This is intriguing since *L. plantarum* does not produce acetate under these conditions. *L. plantarum* has a facultative heterofermentative metabolism, producing large amounts of lactate under anaerobic conditions (43). In addition, we did not observe a decrease in lactate concentration in its absence, indicating that other microorganisms contribute to the lactate pool.

In fecal samples, lactate is not typically detected since it is a cross-feeding metabolite that is readily fermented by other gut microbes (44, 45). In feces from subjects with inflammatory bowel diseases, lactate could reach up to 100 mM (46). In this study, the lowest lactate production was observed by deleting *B. dorei*. *Bacteroides* species are well known for producing acetate and propionate as end products. However, certain studies indicate that they could also release lactate to the medium, for example in the absence of organic nitrogen sources and especially fermentable carbohydrates such as fructans (47, 48). Therefore, we hypothesize that *B. dorei* is the major lactate producer in the consortium. Interestingly, *B. dorei* deletion resulted in the highest acetate concentration obtained and the concomitant dominance of *L. plantarum* (Fig. 3). This observation could be explained by *L. plantarum* switching to heterofermentative metabolism or by another dominant microbe in this consortium, such as E. coli, being responsible for the high acetate observed.

### Mechanistic evidence for metabolic interactions.

Inulin is a well-studied prebiotic that has been generally associated with the stimulation of *Lactobacillus* and *Bifidobacterium* (35, 49, 50). The impact of this fructan in the gut microbiome is regarded to be more complex now, stimulating other species such as *Anaerostipes* and decreasing *Bilophila* (5). In our consortium, at least five species are well known for inulin utilization (*B. adolescentis*, B. thetaiotaomicron, *L. plantarum*, *B. dorei*, and *B. ovatus*), and several others are endowed with fructofuranosidases (*L. symbiosum*, *L. clostridioforme*, *B. cellulosilyticus*, *B. finegoldii*, and *R. gnavus*) (Table 1) (51). Among these, *L. plantarum* and *B. adolescentis* displayed a limited representation in the synthetic consortium, and their growth was usually repressed in the presence of other microbes. Their abundance increased only after prominent members in the consortium were deleted. *B. dorei* or *L. symbiosum* deletion increased *L. plantarum* growth, and E. coli deletion promoted *B. adolescentis*. Our working model shows that *B. dorei* is the dominant microbe accessing inulin and releasing smaller fructans and SCFA, which could support E. coli and *L. symbiosum* growth. How this combination is able to outcompete *L. plantarum* or *B. adolescentis in vitro* is intriguing and could be explained by higher growth rates on the substrate or the release of inhibitory molecules.

Our consortium included seven *Bacteroides* species, including *B. dorei*, discussed above. *Bacteroides* species are important members in the microbiome and are endowed with a wide array of polysaccharide utilization machineries. They are mostly generalists able to explore a broad set of nutrients, including proteins (52). Depending on dietary fibers being fed, *Bacteroides* species could assume distinct metabolic roles, where some species act as primary fermenters accessing complex substrates and releasing smaller degradation products to other species. For example, it was shown that *B. ovatus*, while growing in inulin, releases smaller fructans which are utilized by other *Bacteroides*. *B. ovatus* also supports the growth of B. vulgatus in gnotobiotic mice (53). These two microorganisms showed little representation in this study, which could be explained by a higher inulin-degrading capability of other members of the consortium, such as *B. dorei*. Certain *Bacteroides* species are also sensitive to low pH (54), which could limit their growth in our setup (pH 5.5, simulating the conditions of the proximal colon).

*Lachnoclostridium symbiosum* was a prominent member of this consortium producing butyrate. Although the production of butyrate in *L. symbiosum* occurs preferably via the 4-aminobutyrate/succinate pathway (55), it can also be synthesized from acetate and lactate (56–58). We hypothesize that acetate is being cross-fed to *L. symbiosum*, resulting in butyrate production. While this was not demonstrated here, additional evidence is the presence of homologs of an acetate permease in its genome. Another study suggested that *L. symbiosum* could produce butyrate from lactate (59). Clostridium butyricum is able to use both lactate and acetate for butyrate production (60). *L. symbiosum* was the most sensitive strain with regard to the absence or presence of other microbes. Deletions of E. coli, *B. adolescentis*, *B. dorei*, and *L. plantarum* resulted in limited growth of *L. symbiosum*, suggesting they are important for its growth. A recent report studying the pairwise interactions showed that in a 12-species consortium, Faecalibacterium prausnitzii, a keystone species and butyrate producer, was also the most sensitive species, receiving the most positive interactions from other microbes (17). We have also previously reported that *L. symbiosum*, in the presence of *B. dorei*, produced high concentrations of butyrate which were shown to reduce inflammation mediated by tumor necrosis factor alpha (TNF-α) in a cellular model (48). These positive interactions, which are dependent on diet, could be further exploited in order to enrich the gut microbiome for butyrate-producing species.

### Conclusions.

In this study, we analyzed the impact of the species deletions in a synthetic consortium of gut microbes. This approach was useful to identify significant microbial interactions between microorganisms but also specific microbes playing key metabolic roles in the community. We also observed emergent interactions which cannot be detected using paired cocultures. We observed a strong correlation between E. coli, *B. dorei*, and *L. symbiosum*, and we defined essential species for butyrate production. In addition to identifying key roles in the gut microbiome, this approach could be useful for the design of microbial consortia with desired metabolic properties.

## MATERIALS AND METHODS

### Strains and culture media.

Strains used in this study are shown in Table 1, and were obtained from BEI Resources, the ATCC, or the UC Davis Culture Collection. For routine experiments, microorganisms were cultured in their respective culture media described in Table 1. Luria-Bertani medium (LB; Becton, Dickinson, Franklin Lakes, NJ) was used directly, while reinforced clostridium medium (RCM; Becton, Dickinson, Franklin Lakes, NJ) was supplemented with 0.5 g/liter of l-cysteine (Loba Chemie, India). de Man, Rogosa, and Sharpe medium (MRS; Becton, Dickinson, Franklin Lakes, NJ) was supplemented with 0.5 g/liter of l-cysteine, except for *L. plantarum*. Brain heart infusion (BHI; Becton, Dickinson, Franklin Lakes, NJ) was supplemented with 0.5 g/liter of l-cysteine and 0.01 g/liter of hemin. All incubations were performed at 37°C for 24 to 48 h in an anaerobic jar (Anaerocult; Merck, Darmstadt, Germany) with anaerobic packs (GasPak EM; Becton, Dickinson, Franklin Lakes, NJ).

### Genomic analysis.

Genomes were obtained and analyzed using the Integrated Microbial Gene (IMG) (51). Pairwise average nucleotide identity (ANI) (61) was calculated for genomes used in this study, compared to available finished genomes in the same species. A genetic distance tree was obtained based on the 16S rRNA sequence of each microorganism in IMG. Fructofuranosidase presence in select genomes was estimated using the EC number 3.2.1.26. Inulin utilization and acetate and lactate production were determined from the literature (Table 1) (16). Propionate and butyrate production was confirmed by finding the respective IMG pathways in select genomes. Finally, sporulation was confirmed by the presence of Spo0A homologs and a full set of sporulation genes in select genomes.

### Experimental design and bioreactor operation.

Sixteen batch experiments were carried out in a 250-ml bioreactor connected to a MyControl system (Mini-bio; Applikon Biotechnology, Netherlands). Two of them were inoculated with the complete consortium (All) run in duplicate. The remaining experiments replicated the same conditions as the control with all strains but leaving one species out; therefore, they were inoculated with 13 strains. The bioreactors were named with Δ followed by an abbreviation of the microorganism left out in each case (Table 1). Microorganisms were cultured using the optimized formulation mZMB (62), with a fixed pH of 5.5. Before being inoculated in each experiment, microorganisms were grown individually in mZMB supplemented with lactose (20 g/liter) as a carbon source under anaerobic conditions for 48 h at 37°C. After 48 h, the OD was measured, and the volume required of each culture was considered to use an initial OD_630_ of 1 for each microorganism. Cultures were centrifuged at 3,000 rpm for 5 min. The supernatant was discarded, and the pellet was resuspended in the fixed group of mZMB and used to inoculate the reactor. For each bioreactor, mZMB was supplemented with inulin (20 g/liter) (Piping Rock, Ronkonkoma, NY). Tryptone (Becton, Dickinson, Franklin Lakes, NJ) and l-cysteine were used at 34.2 g/liter and 1 g/liter, respectively, and autoclaved directly in the bioreactor in 70 ml of distilled water. The remaining components of mZMB were sterilized using 0.22-μm filters and incorporated into the bioreactor after being autoclaved (inulin and 0.005 g/liter hemin). The bioreactor was inoculated at an initial OD_630_ of 1 for each microorganism. Foam level was controlled by injecting 200 μl of silicone antifoam (polydimethylsiloxane) with the inoculum. To generate an anaerobic environment inside the bioreactor, nitrogen (99.99% purity grade) was injected at the beginning of the fermentation and remained anaerobic during each experiment. The temperature was set at 37°C, and stirring was set at 90 rpm. pH was maintained constant throughout the fermentation at 5.5, using an automatic injection of 3 M NaOH and 3 M HCl. Samples were taken every 2 h up to 30 h and immediately centrifuged at 10,000 × *g* for 2 min. Pellets and supernatants were stored at −20°C. Pellets were subsequently used for DNA extraction and relative abundance determination, and supernatants were used for quantification of inulin consumption and production of SCFA.

### Substrate consumption.

Inulin utilization was quantified using a previously adapted phenol-sulfuric acid method (62, 63). Supernatants from the bioreactors were diluted 1:200 (vol/vol). Inulin standards between 12 and 0.08 μg were prepared. The experiments were performed in 96-well microplates containing 50 μl of a cold-diluted sample (4°C), 150 μl of concentrated sulfuric acid (98% H_2_SO_4_), and 30 μl of 5% phenol. Microplates were heated at 90°C for 5 min and cooled on ice for 5 min. Absorbance at 490 nm was determined in a Tecan Infinite M200 Pro plate reader (Tecan Trading AG, Grödig, Austria). Measurements were performed in replicates.

### Quantification of SCFA by HPLC.

Acetic, lactic, propionic, and butyric acid were quantified at the end of each run in the bioreactors by high-performance liquid chromatography (HPLC) according to a protocol previously reported (64). An ion exchange column of organic acids and carbohydrates, Aminex HPX-87H (Bio-Rad, Hercules, CA), was used in a Lachrom L-700 HPLC system (Hitachi, Japan) at 35°C with a flow rate of 0.45 ml/min of 5 mM H_2_SO_4_. As standard, concentrations from 30 g/liter to 0.155 g/liter of each acid were used.

### Determination of relative bacterial abundances.

Total DNA extraction from cell pellets was performed by an adapted phenol-chloroform-isoamyl protocol (62). DNA was quantified using a NanoQuant plate in a Tecan Infinite M200 Pro microplate reader and diluted to 10 ng/μl. Bacterial relative and absolute abundances were determined by qPCR, using a set of species-specific primers based on unique genes present in each microorganism (see Table S1 at https://figshare.com/s/a3c67977ccf6fe7292eb). qPCRs and concentration measurements were performed as in the work of Medina et al. (65), with annealing temperatures of 62°C. One exception was *R. gnavus* qPCR, which was performed at 58°C. Reactions were performed in triplicates, and threshold cycle (*C_T_*) values were converted into genome copy numbers per milliliter as in the work of Medina et al. (65).

### Data analysis.

The contribution of microorganisms to the butyrate production was calculated as a linear regression of the abundances (variables) to the production butyrate (response). A single contribution factor was defined by species. The contribution factor was multiplied by the average of the abundance of the respective bacterium determining its contribution. For the multivariate analysis, principal-component analysis and hierarchical clustering were performed with the ClustVis tool (66). An interaction network was built from the Spearman correlation matrix using the ‘corr’ command in Matlab and showing the statistically significant correlations (*P* value < 0.05). Later, we used the bonf_holm function in Matlab (Bonferroni-Holm correction) for multiple testing.

### Data availability.

Raw data and calculations of average nucleotide identity between microorganisms in this study and other strains in the same species are available at https://figshare.com/s/a3c67977ccf6fe7292eb.
